# 1,3,4,6-Tetra­chloro-7,7-bis­(4-chloro­phen­yl)bicyclo­[4.2.0]oct-3-ene-2,5-dione

**DOI:** 10.1107/S1600536808031139

**Published:** 2008-10-22

**Authors:** Huayou Hu, Lei Li, Jun-Feng Ji, Zhi-Guo Shen

**Affiliations:** a School of Chemistry and Chemical Engineering, Southeast University, Nanjing 211189, People’s Republic of China

## Abstract

The title compound, C_20_H_10_Cl_6_O_2_, a quinone derivative, was obtained by the irradiation of 2,3,5,6-tetra­chloro­benzo­quinone and 4,4′-(ethene-1,1-di­yl)bis­(chloro­benzene). The six- and four-membered rings are fused in a *cis* configuration. The dihedral angle between them is 53.4 (3)°.

## Related literature

For related literature, see: Eckert & Goez (1994 ▶); Miyashi *et al.* (1985 ▶); Schenk (1960 ▶); Xu, Song *et al.* (1994 ▶); Xu, Wang *et al.* 1994 ▶); Xue *et al.* (2000 ▶). For a related structure, see: Braun *et al.* (1999 ▶)
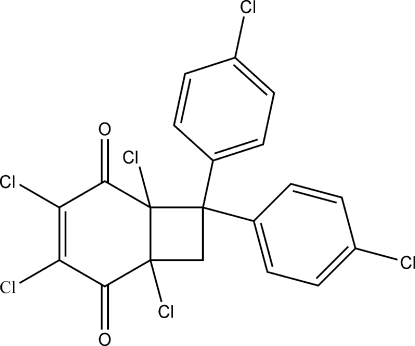


## Experimental

### 

#### Crystal data

C_20_H_10_Cl_6_O_2_
*M* *_r_* = 494.98
Triclinic, 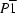

*a* = 8.6710 (17) Å
*b* = 9.6850 (19) Å
*c* = 12.864 (3) Å
α = 105.49 (3)°
β = 97.11 (3)°
γ = 102.68 (3)°
*V* = 996.4 (3) Å^3^
*Z* = 2
Mo *K*α radiation
μ = 0.88 mm^−1^
*T* = 293 (2) K
0.30 × 0.20 × 0.10 mm

#### Data collection

Enraf–Nonius CAD-4 diffractometer
Absorption correction: ψ scan (*SHELXTL*; Sheldrick, 2008 ▶) *T*_min_ = 0.779, *T*_max_ = 0.917
3879 measured reflections
3619 independent reflections
2787 reflections with *I* > 2σ(*I*)
*R*_int_ = 0.049
3 standard reflections every 200 reflections intensity decay: none

#### Refinement

*R*[*F* ^2^ > 2σ(*F* ^2^)] = 0.066
*wR*(*F* ^2^) = 0.192
*S* = 1.00
3619 reflections
253 parameters
H-atom parameters constrained
Δρ_max_ = 0.66 e Å^−3^
Δρ_min_ = −0.57 e Å^−3^

### 

Data collection: *CAD-4 Software* (Enraf–Nonius, 1989 ▶); cell refinement: *CAD-4 Software*; data reduction: *XCAD4* (Harms, 1993 ▶); program(s) used to solve structure: *SHELXS97* (Sheldrick, 2008 ▶); program(s) used to refine structure: *SHELXL97* (Sheldrick, 2008 ▶); molecular graphics: *SHELXTL* (Sheldrick, 2008 ▶); software used to prepare material for publication: *SHELXTL*.

## Supplementary Material

Crystal structure: contains datablocks I, global. DOI: 10.1107/S1600536808031139/bt2797sup1.cif

Structure factors: contains datablocks I. DOI: 10.1107/S1600536808031139/bt2797Isup2.hkl

Additional supplementary materials:  crystallographic information; 3D view; checkCIF report

## Figures and Tables

**Fig. 1.**
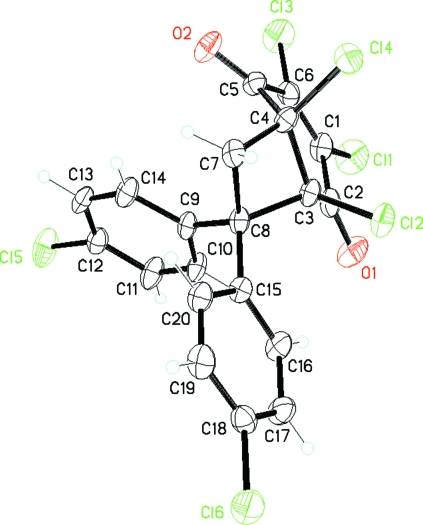
The molecular structure of the title compound with 30% displacement ellipsoids.

## supplementary crystallographic information

### Comment

The reactions of the high potential 2,3,5,6-tetrachlorobenzoquinone with alkenes display varied reaction sites and regioselectivity, depending on the structure of the alkenes and reaction conditions (Schenk 1960; Miyashi *et al.* 1985; Eckert & Goez 1994; Xu, Song *et al.* 1994; Xu, Wang *et al.* 1994). While irradiation of a benzene solution of 2,3,5,6-tetrachlorobenzoquinone and 4,4'-(ethene-1,1-diyl)bis(chlorobenzene) with light of wavelength longer than 400 nm resulted in formation products of the title compound as a yellow solid (Xue *et al.* 2000). The yellow crystals were obtained by recrystallization of these solids from petroleum ether-chloroform.

The title compound, C_20_H_10_Cl_6_O_2_, is a quinone derivative. In the quinone, the distances of the C=O bonds are 1.191 (7) and 1.199 (7) Å, which are considered to to have full double-bond character. Meanwhile, the distances of C1—C2 and C5—C6 are, respectively, 1.478 (9) and 1.475 (8) Å, which are a little longer than that of C1=C6 (1.354 (9) Å), but shorter than those of C—C bonds (1.527 (8)–1.560 (7) Å). This shows that C1—C2 and C5—C6 bonds both have part double-bond character.

### Experimental

Irradiation of a benzene solution of 2,3,5,6-tetrachlorobenzoquinone (0.05 mol *L*_-1_) and 4,4'-(ethene-1,1-diyl)bis(chlorobenzene) (0.10 mol *L*_-1_) with light of wavelength longer than 400 nm for 10 h resulted in complete consumption of 2,3,5,6-tetrachlorobenzoquinone and the formation of products 1,3,4,6-tetrachloro-7,7-bis(4-chlorophenyl)bicyclo[4.2.0]oct-3-ene-2,5-dione. Recrystallization from petroleum ether (bp 60–90 °) and chloroform gave a slightly yellow crystal.

### Refinement

H atoms were positioned geometrically and refined using a riding model with C—H = 0.93–0.97 Å and with *U*_iso_(H) = 1.2 times *U*_eq_(C).

### Crystal data

**Table:**

C_20_H_10_Cl_6_O_2_	*Z* = 2
*M**_r_* = 494.98	*F*(000) = 496
Triclinic, *P*1	*D*_x_ = 1.650 Mg m^−^^3^
Hall symbol: -P 1	Mo *K*α radiation, λ = 0.71073 Å
*a* = 8.6710 (17) Å	Cell parameters from 25 reflections
*b* = 9.6850 (19) Å	θ = 10–13°
*c* = 12.864 (3) Å	µ = 0.88 mm^−^^1^
α = 105.49 (3)°	*T* = 293 K
β = 97.11 (3)°	Block, yellow
γ = 102.68 (3)°	0.30 × 0.20 × 0.10 mm
*V* = 996.4 (3) Å^3^	

### Data collection

**Table:**

Enraf–Nonius CAD-4 diffractometer	2787 reflections with *I* > 2σ(*I*)
Radiation source: fine-focus sealed tube	*R*_int_ = 0.049
graphite	θ_max_ = 25.3°, θ_min_ = 1.7°
ω/2θ scans	*h* = −10→10
Absorption correction: ψ scan (SHELXTL; Sheldrick, 2008)	*k* = −11→11
*T*_min_ = 0.779, *T*_max_ = 0.917	*l* = 0→15
3879 measured reflections	3 standard reflections every 200 reflections
3619 independent reflections	intensity decay: none

### Refinement

**Table:**

Refinement on *F*^2^	Primary atom site location: structure-invariant direct methods
Least-squares matrix: full	Secondary atom site location: difference Fourier map
*R*[*F*^2^ > 2σ(*F*^2^)] = 0.066	Hydrogen site location: inferred from neighbouring sites
*wR*(*F*^2^) = 0.192	H-atom parameters constrained
*S* = 1.00	*w* = 1/[σ^2^(*F*_o_^2^) + (0.06*P*)^2^ + 6*P*] where *P* = (*F*_o_^2^ + 2*F*_c_^2^)/3
3619 reflections	(Δ/σ)_max_ < 0.001
253 parameters	Δρ_max_ = 0.66 e Å^−^^3^
0 restraints	Δρ_min_ = −0.57 e Å^−^^3^

### Special details

**Table:**

Geometry. All e.s.d.'s (except the e.s.d. in the dihedral angle between two l.s. planes) are estimated using the full covariance matrix. The cell e.s.d.'s are taken into account individually in the estimation of e.s.d.'s in distances, angles and torsion angles; correlations between e.s.d.'s in cell parameters are only used when they are defined by crystal symmetry. An approximate (isotropic) treatment of cell e.s.d.'s is used for estimating e.s.d.'s involving l.s. planes.
Refinement. Refinement of *F*^2^ against ALL reflections. The weighted *R*-factor *wR* and goodness of fit *S* are based on *F*^2^, conventional *R*-factors *R* are based on *F*, with *F* set to zero for negative *F*^2^. The threshold expression of *F*^2^ > σ(*F*^2^) is used only for calculating *R*-factors(gt) *etc*. and is not relevant to the choice of reflections for refinement. *R*-factors based on *F*^2^ are statistically about twice as large as those based on *F*, and *R*- factors based on ALL data will be even larger.

### Fractional atomic coordinates and isotropic or equivalent isotropic displacement parameters (Å^2^)

**Table:**

	*x*	*y*	*z*	*U*_iso_*/*U*_eq_	
Cl1	0.3758 (2)	0.1746 (2)	0.61309 (16)	0.0752 (6)	
Cl2	0.0143 (2)	−0.07268 (16)	0.83090 (14)	0.0527 (4)	
Cl3	0.4819 (2)	0.4713 (2)	0.81185 (19)	0.0761 (6)	
Cl4	0.25830 (19)	0.20039 (18)	1.01117 (12)	0.0524 (4)	
Cl5	−0.2014 (3)	0.5682 (2)	0.53637 (17)	0.0788 (6)	
Cl6	−0.7475 (2)	−0.30216 (19)	0.73595 (17)	0.0653 (5)	
O1	0.0754 (6)	−0.0161 (5)	0.6230 (4)	0.0625 (12)	
O2	0.2265 (5)	0.4901 (4)	0.9419 (4)	0.0574 (11)	
C1	0.2697 (7)	0.2059 (7)	0.7168 (5)	0.0444 (13)	
C2	0.1230 (7)	0.0867 (6)	0.7046 (4)	0.0409 (13)	
C3	0.0404 (6)	0.1008 (6)	0.8037 (4)	0.0365 (11)	
C4	0.1223 (7)	0.2309 (6)	0.9110 (4)	0.0382 (12)	
C5	0.2210 (6)	0.3667 (6)	0.8877 (4)	0.0384 (12)	
C6	0.3165 (6)	0.3339 (6)	0.8013 (5)	0.0422 (13)	
C7	−0.0468 (6)	0.2385 (6)	0.9327 (4)	0.0385 (12)	
H7A	−0.0851	0.1819	0.9806	0.046*	
H7B	−0.0580	0.3389	0.9577	0.046*	
C8	−0.1175 (6)	0.1593 (6)	0.8105 (4)	0.0337 (11)	
C9	−0.1295 (6)	0.2656 (6)	0.7408 (4)	0.0360 (11)	
C10	−0.1477 (7)	0.2165 (6)	0.6274 (5)	0.0449 (13)	
H10A	−0.1463	0.1193	0.5932	0.054*	
C11	−0.1680 (9)	0.3087 (7)	0.5634 (5)	0.0554 (16)	
H11A	−0.1766	0.2747	0.4876	0.067*	
C12	−0.1752 (7)	0.4491 (6)	0.6133 (5)	0.0445 (13)	
C13	−0.1599 (7)	0.5022 (6)	0.7252 (5)	0.0452 (13)	
H13A	−0.1630	0.5991	0.7585	0.054*	
C14	−0.1399 (7)	0.4087 (6)	0.7877 (5)	0.0459 (14)	
H14A	−0.1332	0.4432	0.8633	0.055*	
C15	−0.2792 (6)	0.0423 (6)	0.7864 (4)	0.0341 (11)	
C16	−0.3207 (7)	−0.0869 (6)	0.6989 (5)	0.0441 (13)	
H16A	−0.2506	−0.1029	0.6505	0.053*	
C17	−0.4660 (7)	−0.1935 (7)	0.6820 (5)	0.0493 (14)	
H17A	−0.4930	−0.2801	0.6229	0.059*	
C18	−0.5684 (7)	−0.1687 (6)	0.7538 (5)	0.0431 (13)	
C19	−0.5304 (7)	−0.0393 (7)	0.8409 (5)	0.0487 (14)	
H19A	−0.6005	−0.0232	0.8893	0.058*	
C20	−0.3883 (7)	0.0644 (6)	0.8548 (4)	0.0421 (13)	
H20A	−0.3642	0.1527	0.9122	0.050*	

### Atomic displacement parameters (Å^2^)

**Table:**

	*U*^11^	*U*^22^	*U*^33^	*U*^12^	*U*^13^	*U*^23^
Cl1	0.0764 (12)	0.0926 (14)	0.0673 (11)	0.0278 (10)	0.0554 (10)	0.0190 (10)
Cl2	0.0647 (10)	0.0394 (8)	0.0665 (10)	0.0226 (7)	0.0264 (8)	0.0234 (7)
Cl3	0.0535 (10)	0.0653 (11)	0.1098 (16)	0.0045 (8)	0.0405 (10)	0.0250 (10)
Cl4	0.0565 (9)	0.0623 (9)	0.0436 (8)	0.0229 (7)	0.0147 (6)	0.0167 (7)
Cl5	0.1310 (18)	0.0587 (10)	0.0781 (12)	0.0495 (11)	0.0479 (12)	0.0402 (10)
Cl6	0.0518 (9)	0.0553 (10)	0.0947 (13)	0.0112 (7)	0.0326 (9)	0.0267 (9)
O1	0.080 (3)	0.054 (3)	0.049 (3)	0.019 (2)	0.038 (2)	−0.003 (2)
O2	0.069 (3)	0.037 (2)	0.064 (3)	0.014 (2)	0.032 (2)	0.003 (2)
C1	0.050 (3)	0.052 (3)	0.043 (3)	0.025 (3)	0.031 (3)	0.015 (3)
C2	0.048 (3)	0.049 (3)	0.039 (3)	0.030 (3)	0.026 (2)	0.014 (3)
C3	0.048 (3)	0.034 (3)	0.036 (3)	0.019 (2)	0.023 (2)	0.011 (2)
C4	0.047 (3)	0.034 (3)	0.035 (3)	0.013 (2)	0.018 (2)	0.005 (2)
C5	0.036 (3)	0.038 (3)	0.039 (3)	0.010 (2)	0.014 (2)	0.005 (2)
C6	0.032 (3)	0.047 (3)	0.052 (3)	0.014 (2)	0.015 (2)	0.017 (3)
C7	0.047 (3)	0.048 (3)	0.029 (3)	0.023 (2)	0.019 (2)	0.011 (2)
C8	0.042 (3)	0.038 (3)	0.031 (2)	0.021 (2)	0.021 (2)	0.012 (2)
C9	0.044 (3)	0.035 (3)	0.040 (3)	0.020 (2)	0.026 (2)	0.014 (2)
C10	0.067 (4)	0.037 (3)	0.040 (3)	0.028 (3)	0.023 (3)	0.010 (2)
C11	0.091 (5)	0.045 (3)	0.044 (3)	0.033 (3)	0.034 (3)	0.016 (3)
C12	0.047 (3)	0.036 (3)	0.058 (4)	0.014 (2)	0.023 (3)	0.018 (3)
C13	0.055 (3)	0.025 (3)	0.055 (3)	0.013 (2)	0.023 (3)	0.005 (2)
C14	0.060 (4)	0.039 (3)	0.046 (3)	0.021 (3)	0.031 (3)	0.010 (2)
C15	0.041 (3)	0.035 (3)	0.031 (3)	0.012 (2)	0.015 (2)	0.013 (2)
C16	0.048 (3)	0.043 (3)	0.044 (3)	0.015 (2)	0.029 (3)	0.006 (2)
C17	0.046 (3)	0.042 (3)	0.054 (4)	0.011 (3)	0.019 (3)	0.003 (3)
C18	0.038 (3)	0.047 (3)	0.051 (3)	0.014 (2)	0.013 (2)	0.022 (3)
C19	0.049 (3)	0.057 (4)	0.054 (3)	0.024 (3)	0.035 (3)	0.021 (3)
C20	0.050 (3)	0.043 (3)	0.039 (3)	0.020 (3)	0.021 (2)	0.011 (2)

### Geometric parameters (Å, °)

**Table:**

Cl1—C1	1.712 (5)	C9—C14	1.384 (7)
Cl2—C3	1.779 (5)	C9—C10	1.385 (7)
Cl3—C6	1.693 (6)	C10—C11	1.390 (8)
Cl4—C4	1.766 (6)	C10—H10A	0.9300
Cl5—C12	1.740 (6)	C11—C12	1.359 (8)
Cl6—C18	1.734 (6)	C11—H11A	0.9300
O1—C2	1.190 (7)	C12—C13	1.372 (8)
O2—C5	1.200 (6)	C13—C14	1.385 (8)
C1—C6	1.354 (8)	C13—H13A	0.9300
C1—C2	1.478 (8)	C14—H14A	0.9300
C2—C3	1.528 (7)	C15—C20	1.382 (7)
C3—C4	1.560 (7)	C15—C16	1.383 (7)
C3—C8	1.596 (7)	C16—C17	1.393 (8)
C4—C5	1.526 (7)	C16—H16A	0.9300
C4—C7	1.540 (7)	C17—C18	1.370 (8)
C5—C6	1.474 (7)	C17—H17A	0.9300
C7—C8	1.532 (7)	C18—C19	1.383 (8)
C7—H7A	0.9700	C19—C20	1.365 (8)
C7—H7B	0.9700	C19—H19A	0.9300
C8—C15	1.534 (7)	C20—H20A	0.9300
C8—C9	1.545 (7)		
			
C6—C1—C2	123.4 (5)	C14—C9—C10	117.1 (5)
C6—C1—Cl1	121.3 (5)	C14—C9—C8	121.3 (5)
C2—C1—Cl1	115.2 (4)	C10—C9—C8	121.2 (5)
O1—C2—C1	121.5 (5)	C9—C10—C11	121.7 (5)
O1—C2—C3	122.3 (5)	C9—C10—H10A	119.1
C1—C2—C3	116.1 (5)	C11—C10—H10A	119.1
C2—C3—C4	118.3 (5)	C12—C11—C10	119.0 (6)
C2—C3—C8	123.0 (4)	C12—C11—H11A	120.5
C4—C3—C8	86.8 (4)	C10—C11—H11A	120.5
C2—C3—Cl2	106.0 (3)	C11—C12—C13	121.4 (5)
C4—C3—Cl2	110.3 (4)	C11—C12—Cl5	120.6 (5)
C8—C3—Cl2	111.5 (3)	C13—C12—Cl5	118.0 (4)
C5—C4—C7	115.4 (4)	C12—C13—C14	118.8 (5)
C5—C4—C3	112.4 (4)	C12—C13—H13A	120.6
C7—C4—C3	88.4 (4)	C14—C13—H13A	120.6
C5—C4—Cl4	103.3 (4)	C9—C14—C13	121.9 (5)
C7—C4—Cl4	118.9 (4)	C9—C14—H14A	119.0
C3—C4—Cl4	118.7 (4)	C13—C14—H14A	119.0
O2—C5—C6	123.6 (5)	C20—C15—C16	117.9 (5)
O2—C5—C4	121.0 (5)	C20—C15—C8	119.4 (5)
C6—C5—C4	115.2 (5)	C16—C15—C8	122.6 (4)
C1—C6—C5	122.0 (5)	C15—C16—C17	121.0 (5)
C1—C6—Cl3	122.7 (4)	C15—C16—H16A	119.5
C5—C6—Cl3	115.2 (4)	C17—C16—H16A	119.5
C8—C7—C4	89.8 (4)	C18—C17—C16	119.0 (5)
C8—C7—H7A	113.7	C18—C17—H17A	120.5
C4—C7—H7A	113.7	C16—C17—H17A	120.5
C8—C7—H7B	113.7	C17—C18—C19	120.9 (5)
C4—C7—H7B	113.7	C17—C18—Cl6	119.6 (5)
H7A—C7—H7B	110.9	C19—C18—Cl6	119.5 (4)
C7—C8—C15	114.9 (4)	C20—C19—C18	119.0 (5)
C7—C8—C9	114.1 (4)	C20—C19—H19A	120.5
C15—C8—C9	109.7 (4)	C18—C19—H19A	120.5
C7—C8—C3	87.4 (4)	C19—C20—C15	122.0 (5)
C15—C8—C3	117.2 (4)	C19—C20—H20A	119.0
C9—C8—C3	112.1 (4)	C15—C20—H20A	119.0
			
C6—C1—C2—O1	171.0 (6)	C4—C3—C8—C7	−20.4 (4)
Cl1—C1—C2—O1	−6.9 (8)	Cl2—C3—C8—C7	90.1 (4)
C6—C1—C2—C3	−11.0 (8)	C2—C3—C8—C15	100.7 (6)
Cl1—C1—C2—C3	171.1 (4)	C4—C3—C8—C15	−137.3 (4)
O1—C2—C3—C4	173.7 (5)	Cl2—C3—C8—C15	−26.7 (5)
C1—C2—C3—C4	−4.3 (7)	C2—C3—C8—C9	−27.4 (7)
O1—C2—C3—C8	−80.5 (7)	C4—C3—C8—C9	94.6 (4)
C1—C2—C3—C8	101.6 (6)	Cl2—C3—C8—C9	−154.9 (4)
O1—C2—C3—Cl2	49.4 (7)	C7—C8—C9—C14	−25.0 (7)
C1—C2—C3—Cl2	−128.6 (4)	C15—C8—C9—C14	105.5 (6)
C2—C3—C4—C5	29.4 (6)	C3—C8—C9—C14	−122.4 (5)
C8—C3—C4—C5	−96.7 (4)	C7—C8—C9—C10	162.0 (5)
Cl2—C3—C4—C5	151.6 (4)	C15—C8—C9—C10	−67.4 (6)
C2—C3—C4—C7	146.4 (5)	C3—C8—C9—C10	64.7 (7)
C8—C3—C4—C7	20.3 (4)	C14—C9—C10—C11	2.9 (9)
Cl2—C3—C4—C7	−91.5 (4)	C8—C9—C10—C11	176.2 (6)
C2—C3—C4—Cl4	−91.2 (5)	C9—C10—C11—C12	−2.1 (10)
C8—C3—C4—Cl4	142.7 (4)	C10—C11—C12—C13	1.2 (10)
Cl2—C3—C4—Cl4	30.9 (5)	C10—C11—C12—Cl5	−179.7 (5)
C7—C4—C5—O2	44.0 (7)	C11—C12—C13—C14	−1.2 (9)
C3—C4—C5—O2	143.4 (5)	Cl5—C12—C13—C14	179.7 (5)
Cl4—C4—C5—O2	−87.4 (6)	C10—C9—C14—C13	−3.0 (9)
C7—C4—C5—C6	−140.6 (5)	C8—C9—C14—C13	−176.2 (5)
C3—C4—C5—C6	−41.2 (6)	C12—C13—C14—C9	2.2 (9)
Cl4—C4—C5—C6	88.0 (5)	C7—C8—C15—C20	34.1 (7)
C2—C1—C6—C5	−1.7 (9)	C9—C8—C15—C20	−96.0 (5)
Cl1—C1—C6—C5	176.1 (4)	C3—C8—C15—C20	134.7 (5)
C2—C1—C6—Cl3	−178.1 (4)	C7—C8—C15—C16	−145.2 (5)
Cl1—C1—C6—Cl3	−0.3 (8)	C9—C8—C15—C16	84.8 (6)
O2—C5—C6—C1	−155.5 (6)	C3—C8—C15—C16	−44.6 (7)
C4—C5—C6—C1	29.3 (8)	C20—C15—C16—C17	−1.8 (9)
O2—C5—C6—Cl3	21.2 (8)	C8—C15—C16—C17	177.4 (5)
C4—C5—C6—Cl3	−154.1 (4)	C15—C16—C17—C18	0.0 (9)
C5—C4—C7—C8	93.0 (5)	C16—C17—C18—C19	1.0 (9)
C3—C4—C7—C8	−21.1 (4)	C16—C17—C18—Cl6	−178.5 (5)
Cl4—C4—C7—C8	−143.4 (4)	C17—C18—C19—C20	−0.1 (9)
C4—C7—C8—C15	139.6 (4)	Cl6—C18—C19—C20	179.4 (5)
C4—C7—C8—C9	−92.5 (5)	C18—C19—C20—C15	−1.8 (9)
C4—C7—C8—C3	20.7 (4)	C16—C15—C20—C19	2.8 (8)
C2—C3—C8—C7	−142.4 (5)	C8—C15—C20—C19	−176.5 (5)

## References

[bb1] Braun, M., Christl, M., Peters, E.-M. & Peters, K. (1999). *J. Chem. Soc. Perkin Trans. 1*, pp. 2813–2820.

[bb2] Eckert, G. & Goez, M. (1994). *J. Am. Chem. Soc.* **116**, 11999–12009.

[bb3] Enraf–Nonius (1989). *CAD-4 Software* Enraf–Nonius, Delft, The Netherlands.

[bb4] Harms, K. (1993). *XCAD4* University of Marburg, Germany.

[bb5] Miyashi, T., Takahashi, Y., Mukai, T., Roth, H. D. & Schilling, M. L. M. (1985). *J. Am. Chem. Soc.* **107**, 1079–1080.

[bb6] Schenk, G. O. Z. (1960). *Electrochemistry*, **64**, 997–1011.

[bb7] Sheldrick, G. M. (2008). *Acta Cryst.* A**64**, 112–122. 10.1107/S0108767307043930 18156677

[bb8] Xu, J. H., Song, Y. L., Zhang, Z. G., Wang, L. C. & Xu, J. W. (1994). *Tetrahedron*, **50**, 1199–1210.

[bb9] Xu, J. H., Wang, L. C., Xu, J. W., Yan, B. Z. & Yuan, H. C. (1994). *J. Chem. Soc. Perkin Trans. 1*, pp. 571–577.

[bb10] Xue, J., Xu, J.-W., Yang, L. & Xu, J.-H. (2000). *J. Org. Chem.* **65**, 30–40. 10.1021/jo990831r 10813892

